# Multichannel Intraluminal Impedance and pH Monitoring are Complementary Methods in the Diagnosis of Gastroesophageal Reflux Disease in Children

**DOI:** 10.5152/eurasianjmed.2022.20265

**Published:** 2022-02-01

**Authors:** Derya Altay, Tanju Basarır Ozkan, Taner Ozgur, Nilufer Ulku Sahin

**Affiliations:** 1 Department of Pediatric Gastroenterology, Erciyes University School of Medicine, Kayseri, Turkey; 2 Department of Pediatric Gastroenterology, Uludağ University School of Medicine, Bursa, Turkey; 3 Department of Pediatric Gastroenterology, Uludag University School of Medicine, Bursa, Turkey

**Keywords:** Esophageal pH monitoring, gastroesophageal reflux disease, pediatric

## Abstract

**Objective:** Gastroesophageal reflux is considered to be a disease when reflux of gastric contents causes troublesome symptoms in infants and children. The aim of this study was to compare the diagnostic value of the multichannel intraluminal impedance monitoring and only pH monitoring in the diagnosis of gastroesophageal reflux disease in infants and children.

**Materials and Methods:** This prospective cross-sectional study consisted of pediatric patients aged between 1 month and 18 years old with symptoms suggestive of gastroesophageal reflux disease. Patients were divided into 2 groups as younger than 24 months (group 1) and older than 24 months (group 2). Twenty-four hours multichannel intraluminal impedance-pH monitoring was performed on the patients.

**Results:** This study included 50 pediatric patients. The mean age of the patients was 5.35 ± 4.92 years. In group 1, total reflux events were fewer than group 2 (*P *= .03) by pH monitoring. In group 1, the number of non-acid reflux events was higher than in group 2 and in group 2, the number of acidic reflux events was higher than group 1 (*P *= .04). Reflux was detected by multichannel intraluminal impedance-pH monitoring in 13 (40%) of 32 patients who were assessed as negative by pH monitoring.

**Conclusion:** It was concluded that more reliable results were obtained when the 2 methods were used together in this study.

## Introduction

Gastroesophageal reflux (GER) is defined as the retrograde passage of gastric content into the esophagus and is termed as gastroesophageal reflux disease (GERD) if it causes troublesome symptoms and/or complications such as excessive vomiting, failure to thrive, heartburn, coughing, esophagitis, recurrent pneumonia, anemia, dental erosion, or apnea.^1,2^ In the majority of typical cases, a diagnosis of GERD can be made solely by history and physical examination. Further evaluations are recommended in patients with atypical symptoms such as hoarseness, recurrent otitis media, bronchiectasis, apnea, or a life-threatening event.

Esophageal pH monitoring is a method that determines the duration and frequency of esophageal acid exposure. The most important limitation of pH monitoring is its inability to detect non-acidic reflux, especially in infants feeding with breast milk or formulas. Additionally, pH monitoring results are negatively affected by acid-suppressive treatment and acidic beverages. Another limitation of this method is that it fails to detect superimposed acidic reflux episodes.^1^ Multichannel intraluminal impedance (MII) monitoring is superior to pH monitoring for the identification of non-acidic (pH > 7) or weakly acidic reflux (pH 4-7), the physical status of the reflux material (i.e., fluid, gas, or mix), reflux severity, and the clearance and motor functions of the esophagus. This method can also detect the correlations between symptoms and different types of reflux episodes as well as the reflux symptoms associated with acid suppression treatment. On the other hand, MII monitoring is more costly and time-consuming than pH monitoring.^3-5^

In younger infants, including premature infants, and children of all ages, it is particularly difficult to diagnose GERD caused by postprandial reflux and non-acidic reflux via classical pH monitoring. The aim of this study was to compare the diagnostic value of pH monitoring with that of MII-pH monitoring in infants and children.

## Materials and Methods

This prospective cross-sectional study consisted of pediatric patients aged between 1 month and 18 years old in the Pediatric Gastroenterology, Hepatology, and Nutrition outpatient clinic of Uludağ University School of Medicine between January 2014 and May 2014 with symptoms suggestive of GERD.

This study was supported by the Uludağ University Scientific Research Project Unit (KUAP (T)-2013/74) and approved by the Ethics Committee of Uludağ University (Date: June 24, 2014, Approval Number: 2014-13/1). All patients’ parents provided their written informed consent prior to participation.

All patients’ age, presenting complaints, and comorbid conditions were questioned. As regurgitation lasts up to 24 months of life in infants, the patients included in the study were divided into 2 groups according to age: group 1, aged <24 months (n = 20, 40%); and group 2, aged >24 months (n = 30, 60%). Pediatric intensive care unit patients with poor general status and the patients who had not withdrawn antacid and prokinetic agents for at least 7 days were excluded from the study.

Multichannel intraluminal impedance-pH monitoring was performed for all patients, and then MII-pH and only pH monitoring results were analyzed separately. Antacid and prokinetic agents were withdrawn at least 7 days prior to MII-pH monitoring in order to prevent confounding effects. Before testing began, the recording devices and catheters were calibrated using solutions with pH values of 7 and 1 (Reagecon pH buffer solutions), respectively. After the patients had fasted for 6 hours, a disposable catheter (1.5 mm thick, Unisensor K6011-EI-0633 catheter) carrying 6 impedance sensors and a pH sensor was inserted into the esophagus of each patient through the nasal route. Chest radiographs were obtained to confirm that the catheters were positioned correctly. Each catheter was attached to a recording device (MMS Ohmega Ambulatory Impedance and pH recorder, the Netherlands). Patients and/or relatives were asked to maintain normal dietary habits and routine daily life during the procedure. Patients and/or relatives were also asked to record the timing of nutrition, symptoms (coughing, heartburn, regurgitation of food or sour liquid, etc.) and changes in position (e.g., supine or standing) using pushbuttons on the MII-pH device. The recording period was set for 24 hours of continuous recording over the course of 1 night.

After the procedure was completed, the data were transferred to a desktop PC and analyzed using an MMS Investigation and Diagnostic Software. To assess pH monitoring, the following parameters were calculated: the reflux index (the ratio of duration at pH < 4 to total recording time), the number of reflux episodes per 24 hours, the number of reflux episodes lasting longer than 5 minutes per 24 hours, the longest reflux period, and the lowest pH parameter.

For the MII assessment, the onset of a reflux episode was defined as an acute decrease in basal esophageal impedance by 50% in at least 2 channels, from distal to proximal, whereas cessation of the reflux episode was defined as the point at which the impedance value reached at least 50%. For pH monitoring, a positive outcome was defined by a reflux index >6%. For MII monitoring, a positive outcome was defined by >70 reflux episodes per day in patients aged ≥1 year or by >100 reflux episodes per day in patients aged <1 year.^6,7^

## Statistical Analysis

Data were analyzed using Statistical Package for the Social Science (SPSS) for Windows version 16 (SPSS Inc.; Chicago, IL, USA) and MedCalc version 18.11. A Pearson chi-square test was used to assess the differences in the frequencies of the categorical variables. A Shapiro–Wilk test was used to assess the normal distribution. A Mann–Whitney *U*-test was used to compare the differences in the median values of the continuous variables between groups. Cohen’s kappa agreement test was also used to determine the agreement of the impedance and esophageal pH monitoring to detect gastroesophageal reflux. According to Cohen’s kappa agreement test, kappa: <0.00 means poor agreement, kappa: 0.00-0.20 means slight agreement, kappa: 0.20-0.40 means slight fair agreement, kappa: 0.41-0.60 means moderate agreement, kappa: 0.61-0.80 means substantial agreement, and kappa: 0.81-1.00 means almost perfect agreement. A *P*-value <.05 was considered to be statistically significant.

## Results

The participant sample of the present study included 50 children who presented to the Pediatric Gastroenterology outpatient clinic of Uludağ University School of Medicine with nausea, vomiting, failure to thrive, feeding difficulties, loss of appetite, abdominal pain, coughing, retrosternal burn sensation, halitosis, and frequent respiratory tract infections between January and May 2014. This sample included 26 females (52%) and 24 males (48%). The mean age at presentation was 5.35 ± 4.92 years (with a range of 2.5 months-17 years). While patients in the younger age group mostly presented with vomiting symptoms, heartburn and regurgitation were more prominent in the older age group. Table 1 presents demographic data and the most common presenting complaints in the participant groups.

Based on the evaluation of cases according to pH monitoring results, the total reflux count (per 24 hours) of group 1 was significantly lower than group 2 (*P *= .03). However, no difference was found between the groups in terms of reflux index, the number of reflux lasting more than 5 minutes, the longest reflux period, and the lowest pH parameter (*P *> .05). Table 2 shows the comparison of the 2 groups’ 24-hour pH monitoring results.

On the other hand, when the cases were evaluated according to MII-pH monitoring, the number of acidic reflux episodes in group 2 was significantly higher than group 1 (*P *= .04), and the weakly acidic reflux number in group 1 was higher than group 2 (*P *= .03). However, no statistically significant difference was found between the groups in terms of reflux numbers, non-acid reflux numbers, liquid reflux, and mixed reflux (*P *> .05). A comparison of the number of the acidic, weakly acidic, non-acidic, liquid, and mixed refluxes of groups is shown in Table 3. According to the study, the results of reflux detection through MII-pH or pH monitoring were as follows; pH monitoring detected only 8 patients amongst 21 patients who were detected reflux by MII-pH. While MII-pH detected only 8 patients amongst 18 patients who were detected reflux by pH monitoring. Therefore, only 8 patients were diagnosed with GERD by both methods. Also noted that neither of the methods could not detect any reflux episodes in 19 of 50 patients (*P *> .05). In group 1, reflux was detected in 4 cases with both pH and MII-pH monitoring, while reflux was not detected in 7 cases with either method (*P *> .05). While reflux was detected by both methods in 4 cases in group 2, reflux was not detected with either method in 12 cases (*P *> .05). An agreement of the reflux detection ratios yielded by MII-pH monitoring or pH monitoring for each group is shown in Tables 4 and 5. No agreement was detected between the 2 methods.

There were 4 preterm (28 weeks of gestation) infants in the present sample; 2 patients were aged 6 months (corrected age, 3 months), 1 patient was aged 19 months, and 1 patient was aged 38 months. One of the 2 patients aged 6 months was deemed positive for reflux by pH monitoring and negative for reflux by MII monitoring, while the remaining patient was deemed positive for reflux by MII monitoring and negative for reflux by pH monitoring. The patient aged 19 months was deemed negative for reflux by both pH and MII monitoring, while the patient aged 38 months was deemed positive for reflux by MII monitoring and negative for reflux by pH monitoring. Two patients with hypoxic-ischemic encephalopathy were deemed positive for reflux by MII monitoring. There were 10 patients in total with cough complaints. One of the coughing cases in group 1 had recurrent pulmonary infection. While reflux was not detected with pH monitoring in 10 cases, weakly acidic reflux was found with the MII-pH method in 2 cases, one of whom had recurrent pulmonary infection.

Refluxes reaching the proximal part of the esophagus were detected in 13 cases (26%) and in 7 of these cases (53.8%), the refluxes were weakly acidic.

## Discussion

Esophageal pH monitoring has been accepted as the gold standard of GERD diagnosis methods for many years. However, this method had begun to fall out of favor by 2002, following the introduction of combined MII-pH monitoring in pediatric practice; the existence of this trend has been supported by several studies.^8-12^ Gastroesophageal reflux disease can present with various clinical manifestations, including esophageal (i.e., vomiting, dysphagia, or esophagitis) and extra-esophageal (i.e., respiratory complaints) symptoms.^13^ In most symptomatic infants, GER resolves by 12–24 months of age.^14^ Therefore, the cases included in the study were divided into 2 groups as those younger than and older than 24 months. In the present study, presenting complaints included nausea, vomiting, failure to thrive, feeding difficulties, coughing, chest pain or heartburn, recurrent pulmonary infections, halitosis, and abdominal pain. The most common complaints of patients aged <24 months were vomiting, inadequate weight gain, and coughing, whereas vomiting, heartburn, regurgitation of food or sour liquid, coughing, and nausea were most common for patients aged >24 months.

For pH monitoring, reflux episodes are defined by pH values <4; thus, weakly acidic or non-acid refluxes cannot be detected by this method. Given that approximately 90% of reflux episodes in infants are non-acidic due to frequent feeding, pH monitoring has limited value for this group.^9^ In this study, the number of weakly acid reflux was higher in the infantile group compared to the older age group. No non-acid reflux was detected in either group.

Multichannel intraluminal impedance monitoring is a novel method that is considered to be the most reliable test used for GERD diagnosis according to numerous preexisting studies.^7,15-20^ Of the 50 patients included in the present sample, 21 (42%) were deemed positive for reflux by MII-pH monitoring and 18 (36%) were deemed positive for reflux by pH monitoring. In a study by Francavilla et al^9^ were suggested that combined MII-pH monitoring is a potent first-line diagnostic method for children with GERD.

In the present study, the reflux index (defined as the ratio of time during which pH was <4 to total reflux time) was not statistically different between the groups, but the number of reflux episodes per 24 hours was significantly lower in group 1 compared to group 2 (*P *= .03). This finding was attributed to the higher rate of weakly-acid reflux in group 1 compared to group 2. However, 24-hour pH monitoring only detects acid reflux events.

Gastroesophageal reflux-related complications are seen more frequently in newborns and infants since their anti-reflux mechanisms are not sufficiently developed.^2^ Preterm infants are particularly prone to developing GER for a variety of reasons, including their tendencies to always be in supine positions, have short and narrow esophagus, have lower esophageal sphincters just above the diaphragm, and engage in frequent or high volume feeding by milk or formula. Similar to term infants, premature infants tend to see a decrease in GER incidence as they become more mature.^21-23^

In this study, 2 of the 4 patients who were born preterm had weakly acidic reflux detected by MII-pH, and 1 patient had acid reflux detected by pH monitoring. One of the patients with weakly acidic reflux was a 6-month-old infant and this was an expected situation because the patient was fed with the formula frequently. However, the other patient with weakly acidic reflux was 38 months old, and preterm-related GER was already resolved in this patient. The growth of this case was normal and the patient was admitted with coughing. Laura et al^24^ reported that chronic cough was associated with weakly acidic reflux in children with MII-pH. In this study, acid reflux was found in a 6-month-old preterm infant, and acid reflux was attributed to the regurgitation of stomach contents in this patient, whose corrected age was three months old and presented with vomiting. Due to the small number of preterm patients, sufficient results could not be achieved.

The risk for GERD is higher in children with growth retardation or neurological disorders compared to children with normal development. This difference can be attributed to differences in neuromuscular coordination, lower esophageal sphincter dysfunction, or esophageal motility disorder.^25,26^ Çaltepe et al^27^ reported that children with cerebral palsy are often fed with liquid foods, with the vast majority of reflux being weakly acid or non-acid reflux. The present study found that reflux was detected by MII monitoring but not by pH monitoring in 2 patients with hypoxic-ischemic encephalopathy, thus suggesting that weakly acidic reflux or non-acid reflux could have been caused by esophageal motor dysfunction.

In GERD, extra-esophageal symptoms such as persistent cough or asthma result from the direct deleterious effects of gastric fluid on the upper and lower airways. Since there are no anti-reflux clearance mechanisms in airways such as the distal esophagus, even a single reflux episode reaching the proximal esophagus can cause symptoms.^28^ In the present study, 2 patients from group 1 and 8 patients from group 2 exhibited coughing. One of the 2 patients in group 1 exhibited recurrent pulmonary infections. Ten patients (20%) were deemed negative for reflux by pH monitoring. Reflux was detected by MII-pH in only 2 patients. The patient with recurrent pulmonary infections was deemed negative for reflux by pH monitoring but was deemed positive for reflux by MII-pH monitoring; this finding suggests that MII-pH monitoring can be used as a complementary test in symptomatic patients.

It was determined that using MII and pH monitoring combined can allow for the detection of all acidic and non-acidic refluxes and can be used to determine the contents of the reflux, the level to which the reflux reached, and parameters such as relationship with symptoms.^29,30^ Using MII-pH monitoring, the present study showed that one-quarter of all refluxes reached up to the proximal esophagus and that half of these refluxes were weakly acidic.

Combined MII-pH monitoring is a sensitive method for the assessment of non-acidic refluxes in particular. Wenzl et al^31^ reported that 78% of GER reflux episodes causing respiratory derangement in infants were non-acidic. In a study of 28 children with respiratory symptoms, Rosen and Nurko^32^ reported that 45% of reflux episodes were non-acidic. In a study of 106 children with persistent coughing, Ghezzi et al^33^ used MII-pH monitoring to determine that both weakly acidic reflux and acidic reflux could lead to coughing attacks in younger children. The present study found that reflux detected by MII-pH monitoring in patients with coughing was weakly acidic reflux.

The limitation of the study was that the number of patients with special conditions such as preterm birth and hypoxic-ischemic encephalopathy was low. Therefore, the interpretation of the results was limited. In addition, any method in this study was not accepted as the gold standard.

The present study showed that MII-pH monitoring, a novel method for GERD diagnosis with increasing popularity, is especially effective in the detection of weakly acidic reflux during the infantile period. It is considered that it will be used in the diagnosis of GERD more widely in the future since it can detect reflux types that cannot be detected by pH monitoring alone. However, the use of this method is restricted because it is expensive, time-consuming, and has limited pediatric reference values. The present study compared MII-pH and pH monitoring of children with suspected GERD, and it was concluded that combined use of these methods provided more reliable results.

## Figures and Tables

**Table 1.** Demographic Data of the Patients and Comparison of the Most Common Symptoms of the 2 Groups

Age (Mean+SD, Months) Gender (Male/Female)	Group 1 (n = 20)	Group 2 (n = 30)	*P*
10.9+7.2 9/11	99.0+52.1 15/15
Vomiting Failure to thrive Cough Heartburn Regurgitation of food or sour liquid Nausea	17 7 2 0 0 0	8 3 8 8 8 6	<.001.05 .31 .01 .01 .63

**Table 2.** Comparison of the Results of 24-hour pH Monitoring Parameters of the 2 Groups

Results	Group 1 [Median (Interquartile Range)]	Group 2 [Median (Interquartile Range)]	*P*
Reflux index (%)	0.72 (0.04-3.41)	1.47 (0.69-2.75)	.69
Number of reflux per 24 hours	10 (2.5-17.25)	19 (7-35)	.03
Number of reflux longer than 5 minutes	0 (0-1)	0 (0-1.25)	.58
Longest reflux period (minutes)	2.85 (0.55-14.30)	3.70 (1.55-11.67)	.73
Lowest pH parameter	1.85 (1.45-2.32)	1.40 (1.17-1.95)	.57

**Table 3.** Comparison of the Results of MII-pH Monitoring Parameters of the 2 Groups

Results	Group 1[Median (Interquartile Range)]	Group 2 [Median (Interquartile Range)]	*P*
Number of reflux	44 (29-74)	36 (15-54.25)	.26
Number of acidic reflux (pH<4)	5 (0.75-15.75)	13.5 (3.75-27.50)	.04
Number of weakly acidic reflux (4<pH<7)	27.50 (14.25-45.75)	19 (8.50-27.75)	.03
Number of non-acid reflux (pH>7)	0 (0-4.74)	0 (0-2)	.05
Liquid reflux	8.50 (2.75-22.75)	6.50 (4-14.5)	.56
Mixed reflux	30.5 (19.25-46.75)	27.5 (10.25-43.75)	.83

**Table 4.** Agreement of the Reflux Detection Ratio by MII-pH Monitoring or pH Monitoring of the Patients in Group 1

	MII-pH Monitoring +	MII-pH Monitoring −
pH monitoring +	4 (20%)	3 (15%)
pH monitoring −	6 (30%)	7 (35%)

+, Reflux detected, −, Reflux not detected, Kappa = 0.1, *P *= .63.

Positive predictive value = 57.1%, Negative predictive value = 53.8%

**Table 5.** Agreement of the Reflux Detection Ratio by MII-pH Monitoring or pH Monitoring of the Patients in Group 2

	MII-pH monitoring +	MII-pH monitoring −
pH monitoring +	4 (13.3%)	7 (23.3%)
pH monitoring −	7 (23.3%)	12 (40%)

+, Reflux detected, −, Reflux not detected, Kappa < 0.005, *P *= .97.

Positive predictive value = 36.6%, Negative predictive value = 63.1%
